# Female-to-male sex conversion in *Ceratitis capitata* by CRISPR/Cas9 HDR-induced point mutations in the sex determination gene *transformer-2*

**DOI:** 10.1038/s41598-020-75572-x

**Published:** 2020-10-29

**Authors:** Roswitha A. Aumann, Irina Häcker, Marc F. Schetelig

**Affiliations:** Department of Insect Biotechnology in Plant Protection, Institute for Insect Biotechnology, Justus-Liebig-University Gießen, Winchesterstr. 2, 35394 Giessen, Germany

**Keywords:** Agricultural genetics, Genetic engineering, Mutagenesis

## Abstract

The Sterile Insect Technique (SIT) is based on the mass release of sterilized male insects to reduce the pest population size via infertile mating. Critical for all SIT programs is a conditional sexing strain to enable the cost-effective production of male-only populations. Compared to current female-elimination strategies based on killing or sex sorting, generating male-only offspring via sex conversion would be economically beneficial by doubling the male output. Temperature-sensitive mutations known from the *D. melanogaster transformer-2* gene (*tra2*^*ts*^) induce sex conversion at restrictive temperatures, while regular breeding of mutant strains is possible at permissive temperatures. Since *tra2* is a conserved sex determination gene in many Diptera, including the major agricultural pest *Ceratitis capitata*, it is a promising candidate for the creation of a conditional sex conversion strategy in this Tephritid. Here, CRISPR/Cas9 homology-directed repair was used to induce the *D. melanogaster-*specific *tra2*^*ts*^ SNPs in *Cctra2*. 100% female to male conversion was successfully achieved in flies homozygous for the *tra2*^*ts2*^ mutation. However, it was not possible, to identify a permissive temperature for the mutation allowing the rearing of a *tra2*^*ts2*^ homozygous line, as lowering the temperature below 18.5 °C interferes with regular breeding of the flies.

## Introduction

The production of large populations of only male pest insects is a key factor for the Sterile Insect Technique (SIT), a highly successful, environment-friendly, and species-specific method of pest control. Proposed in 1955 by Knipling^1^, the SIT is based on the sustained mass-release of sterile males into the existing pest population to reduce population size by infertile mating, and has been successfully applied to several pest species^2–5^. The release of pure male populations is important because male-only releases are more effective than bisexual ones^6^ by preventing the mating of sterile males with the co-released sterile females. In addition, the release of sterile females could still result in crop damage due to oviposition or, in case of vector insects, in disease transmission. Sexing, classified as the removal of females from a mass-reared insect population, can be achieved by physical sorting, female-specific lethality, or by converting females into males^7^. Such solutions have been developed for multiple pest species using naturally occurring or classically induced mutations^8–11^ or transgenesis^12–17^. Most of them, however, are not ready for mass-rearing yet. To allow efficient rearing of sexing strains and cost-effective operation of the program, important characteristics of sexing systems are the conditionality and early developmental time-point of the sexing, respectively. Currently, two conditional embryonically active systems exist for the devastating agricultural pest *Ceratitis capitata* (Wiedemann; Diptera: Tephritidae) (Mediterranean fruit fly, medfly). Medflies pose a vast economic threat to agriculture worldwide, as they feed on > 260 plants (fruits, vegetables, nuts) and are highly invasive: Native to the Afrotropical region, medfly can now be found in most tropical and temperate regions^18,19^.

In the successful medfly genetic sexing strains (GSS), VIENNA 7 and 8, an unknown recessive autosomal temperature-sensitive lethal (*tsl*) mutation eliminates all female embryos upon heat shock^11^. The GSS males, however, are semi-sterile due to chromosomal rearrangements necessary to rescue the WT phenotype, resulting in 50% genetically imbalanced gametes and thus non-viable zygotes. In a conditional transgenic embryonic sexing system (TESS) medfly female embryos are killed by overexpression of a pro-apoptotic gene^20^. The TESS can be switched off for strain maintenance by adding the antibiotic tetracycline to the fly food (Tet-off system). Compared to these systems, a sexing system based on temperature-inducible female-to-male conversion would have two advantages: (1) doubling or, compared to semi-sterile GSS, even quadrupling the number of males for the release and (2) abolishing the use of antibiotics. Both factors would considerably reduce costs and increase the efficiency of a medfly SIT program. However, population maintenance would presumably need to be done at reduced temperatures, which could decrease the productivity of the mass-rearing due to prolonged development times^21^. Currently, the production of one million sterile medfly pupae of the classical GSS is estimated at US$ 250–500, depending on the production level and the location of the rearing facility^22^.

In search of genetic elements suitable to construct sexing or sex-conversion systems, insect sex determination pathways have been studied to identify essential genes and to understand their function. The *transformer-2* gene (*tra2*) is involved in the sex determination pathway of different insects, including *C. capitata*^23,24^. In medfly, *transformer-2* is an auxiliary factor, necessary to establish and sustain the autoregulation of *transformer*, a gene known to be crucial for the sexual fate^23,25,26^. As illustrated and described in detail elsewhere^23,26^, maternal *Cctra* and *Cctra2* initiate a positive feedback loop in XX fertilized eggs and control the female-specific splicing of the downstream targets *doublesex* and *fruitless*^23,26^. Switching off either *Cctra* or *Cctra2* leads to male development^26^ and the transient knock-down of *Cctra2* during embryogenesis via RNA interference (RNAi) resulted in full sex-reversal of XX-karyotype flies into phenotypic males^23^. In contrast to *Anastrepha suspensa*, where embryonic injection of dsRNA against *Astra2* resulted in sex-reversed XX males, which were infertile despite testes full of sperm bundles^27^, medfly XX-karyotype males were fertile^23,25^, indicating that male-fertility is not Y-dependent in *C. capitata*. Sex-reversion via RNAi-mediated knock-down of *tra2* was also shown in *Bactrocera tau* (Walker)^28^ and *B. dorsalis* (Hendel)^29^. However, to make use of the *tra2*-mediated sex-conversion for male-only production, it needs to be conditionally inducible and stable. In *Drosophila melanogaster,* two *tra2* temperature-sensitive mutations (*tra2*^*ts1*^*, tra2*^*ts2*^) are known, supposedly causing conformational changes in the protein structure at elevated (restrictive) temperatures (29 °C). These result in a loss of protein function and therefore in sex-conversion of XX embryos (male-only offspring). At permissive temperatures (e.g. 16 °C), a functional TRA2 protein allows healthy female development and rearing of the population^30,31^. Due to the high conservation of TRA2 among different species^23,32–36^, gene editing techniques such as CRISPR/Cas^37^ can be used to exactly recreate temperature-sensitive *tra2* mutations known from *D. melanogaster* in homologous genes of pest insects. This has been shown by Li and Handler^38^, who introduced the *D. melanogaster tra2*^*ts2*^ mutation together with a fluorescent marker into the *D. suzukii tra2* gene*.* 16 °C and 20 °C were permissive temperatures for *D. suzukii tra2*^*ts2*^ mutants^38^, resulting in fertile and normally developed males and females. At 26 °C, all XX embryos developed as sterile intersex with sex combs and male-like genitalia, and all XY embryos showed dysmorphic testes and were sterile. However, the survival rate for both, wild-type and mutant flies was very low (5–10%) at this temperature and even lower at more elevated temperatures. While this temperature-sensitivity of *D. suzukii* would be problematic if the *tra2*^*ts2*^ mutation were to be used for sexing in an SIT application, this should not be an issue for medfly, which can be reared at 26 °C. Based on this fact and the promising results from the previous transient knock-down of *tra2* in *C. capitata*^23^, *Cctra2* is a good candidate for the construction of a temperature-based sex-conversion system in medfly.

Hence, we used our previously established protocol for markerless CRISPR/Cas9-HDR in medfly yielding high-efficiency mutagenesis^39^ to integrate the *D. melanogaster tra2*^*ts1*^ and *tra2*^*ts2*^ mutations into the *Cctra2* homolog. Omitting the use of a fluorescent marker gene should facilitate the use of non-transgenic strains in SIT programs, as CRISPR/Cas9-induced single nucleotide polymorphisms (SNP) are even considered non-GMO in certain countries^40^.

## Results

### *Cctra2* mutagenesis: gRNA and repair template design

**Figure 1:**
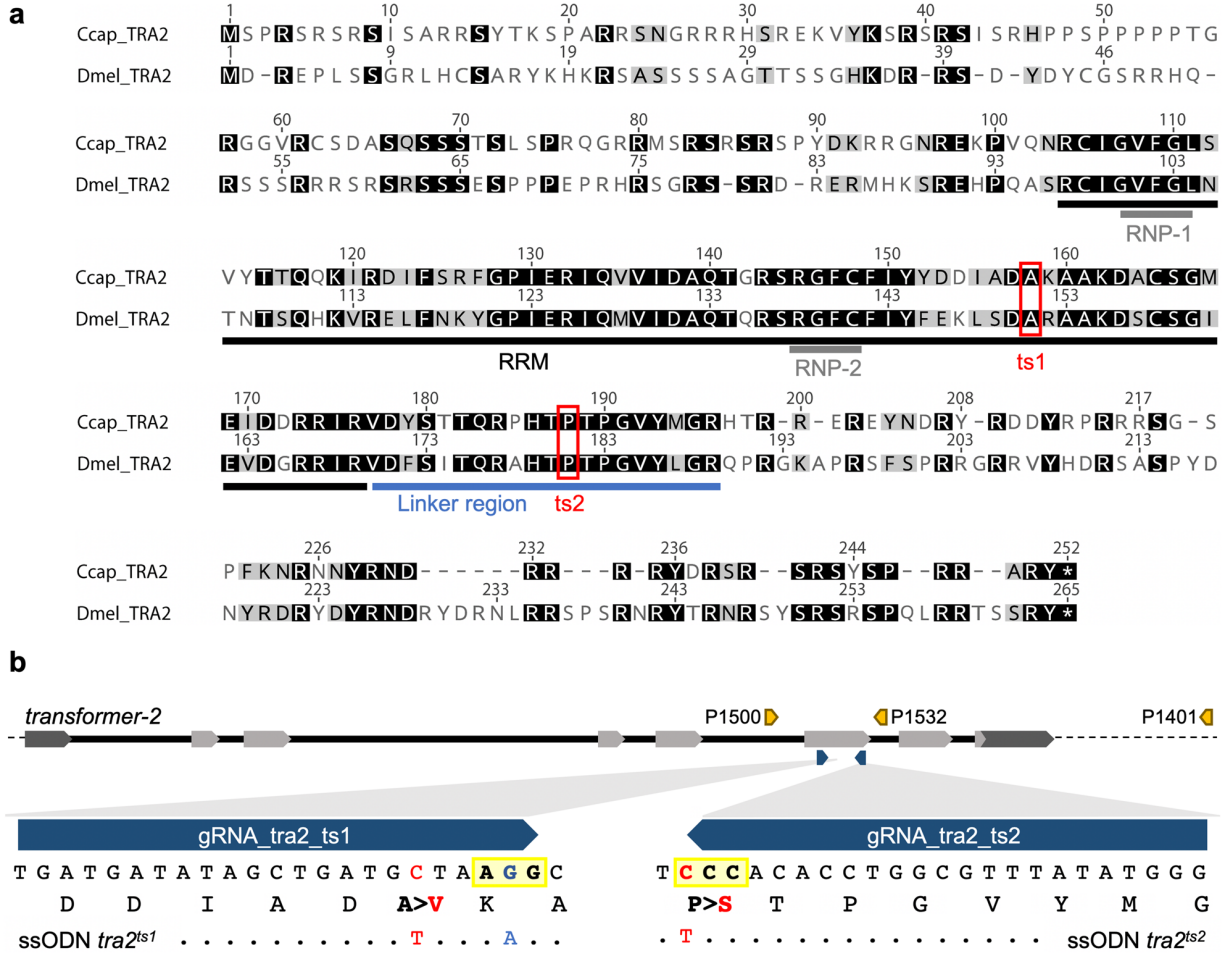
Strategy to re-create *D. melanogaster tra2*^*ts*^ alleles in *C. capitata tra2*. (**a**) Amino acid alignment of *C. capitata* and *D. melanogaster* TRA2. Shown are the RNA recognition motif (RRM, black), two ribonucleoprotein identifier sequences (RNP motifs, grey), the linker region (blue), and the position of the *tra2*^*ts1*^ and *tra2*^*ts2*^ mutations (red). Consensus is shown in black, amino acids with similar characteristics in grey. (**b**) Overview of *Cctra2* gene structure (*tra2* exon structure, light grey: CDS, dark grey: UTR), primers used for genotyping (P1500/P1401) or for genomic positive control PCRs (P1500/P1532 and P1500/P1401), position of single guide RNAs (blue arrows) and mutations mediated by the HDR repair templates (ssODN). PAM sequences are marked in light yellow, position of SNPs introduced by HDR are shown and marked either in blue (silent mutation) or in red (functional mutations). Resulting amino acid exchanges are indicated.

CRISPR/Cas9 HDR gene editing was used to separately re-create the two temperature-sensitive *D. melanogaster tra2* mutations (*ts1*, *ts2*) in the *C. capitata* homolog *Cctra2* (NCBI Gene ID: 101452698). Positions of the mutations were determined by comparing amino acid sequence identity for *D. melanogaster* and medfly TRA2. The mutated Alanine151 in the *Dmel tra2*^*ts1*^ (Ala151Val)^30^ corresponds to *Ccap* Ala158, the Prolin181 of the *Dmel tra2*^*ts2*^ mutation (Pro181Ser) to *Ccap* Pro188. The *ts2* mutation is located in a 19 aa linker region, which is a unique feature of TRA2 and highly conserved among species^23,32–36^ (Fig. 1a).

For both mutations, a single guide RNA (gRNA) and a 140 nt single-stranded oligodeoxynucleotide (ssODN) repair template were designed to introduce the amino acid exchanges corresponding to the *Dmel ts1* or *ts2* mutations (*ts1*: 158 Ala > Val, *ts2*: 188 Pro > Ser), to create temperature-sensitive versions of the CcTRA2 protein. The repair template ssODN_tra2_ts1 differs from the wild-type *tra2* ORF sequence by two bases, a C > T transition at position 473 of the CDS to introduce the *ts1* SNP and the silent mutation 477 G > A that removes the PAM sequence to prevent re-editing. ssODN_tra2_ts2 differs by one base introducing the *ts2* SNP (CDS: 562 C > T) (Fig. 1b).

### Preliminary gRNA tests to confirm editing capability of *tra2*^*ts*^ positions

To assess the functionality of the *tra2*^*ts1*^ and *tra2*^*ts2*^ gRNAs, each was injected complexed with Cas9 protein and either without (non-homologous end joining, NHEJ, knock-out) or with repair template (homology-directed repair, HDR, knock-in). G_0_ survivors of these injections were reared at 26 °C. 327 *Egypt II* wild-type (*EgII* WT) embryos were injected for tra2_ts1 knock-out. Ten reached adult stage (six males, four females) (Table 1a). One male was fertile. The *ts1* injection with repair template (290 *EgII* embryos) yielded four viable but infertile adults (two males, two females), and three adults got stuck in the puparium while eclosing and died (two males, one female) (Table 1a). None of the *ts1* G_0_ adults showed external phenotypic abnormalities. To check for editing activity of gRNA_tra2_ts1, the *tra2* genotype of four randomly chosen G_0_ flies (two from each injection) was analysed by subcloning the *tra2-*specific PCR products. One of two knock-out injected G_0_ flies showed a 1 bp deletion in one of five sequenced clones. One of the knock-in injected G_0_ flies showed two independent events within five sequenced clones, the *tra2*^*ts1*^ HDR genotype or a 6 bp deletion (Supplementary Fig. S1a).

**Table 1:** Summary of injections for targeted *Cctra2* knock-out or knock-in mutations.

	26 °C, KO	26 °C, KI	19 °C, KI
(a) Injections for target *tra2*^*ts1*^			
Injected embryos	327	290	169
Larvae (% hatching)	32 (9.8%)	28 (9.6%)	55 (32.5%)
Pupae	16	8	42
G_0_ adults viable; not viable (% eclosion)	10;0 (3.0%)	4;3 (2.4%)	33;0 (19.5%)
G_0_ viable males (fertile)	6 (1)	2 (0)	11 (> 4)
G_0_ viable females (fertile)	4 (0)	2 (0)	22 (> 2)
G_0_ viable intersex (fertile)	0	0	0

(b) Injections for target *tra2*^*ts2*^			
Injected embryos	367	244	181
Larvae (% hatching)	29 (7.9%)	30 (12.3%)	52 (28.7%)
Pupae	12	18	17
G_0_ adults viable; not viable (% eclosion)	6;3 (2.4%)	8;4 (4.9%)	11;2 (7.1%)
G_0_ viable males (fertile)	6 (3)	6 (6)	5 (2)
G_0_ viable females (fertile)	0	0	0
G_0_ viable intersex (fertile)	0	2 (0)	6 (0)

Shown is the mutation target *tra2*^*ts1*^**(a)** and *tra2*^*ts2*^ **(b)**, the strategy (knock-out (KO) or knock-in (KI)), the rearing temperature, the number of injected embryos and surviving G_0_ larvae, pupae and adults, the larval and adult hatch rate, and the number, phenotypic sex and fertility of viable G_0_ adults. Number of fertile flies for the *tra2*^*ts1*^ KI injection at 19 °C could not be exactly assessed, as only twelve flies were backcrossed individually and remaining 21 flies were backcrossed in three groups.

**Figure 2:**
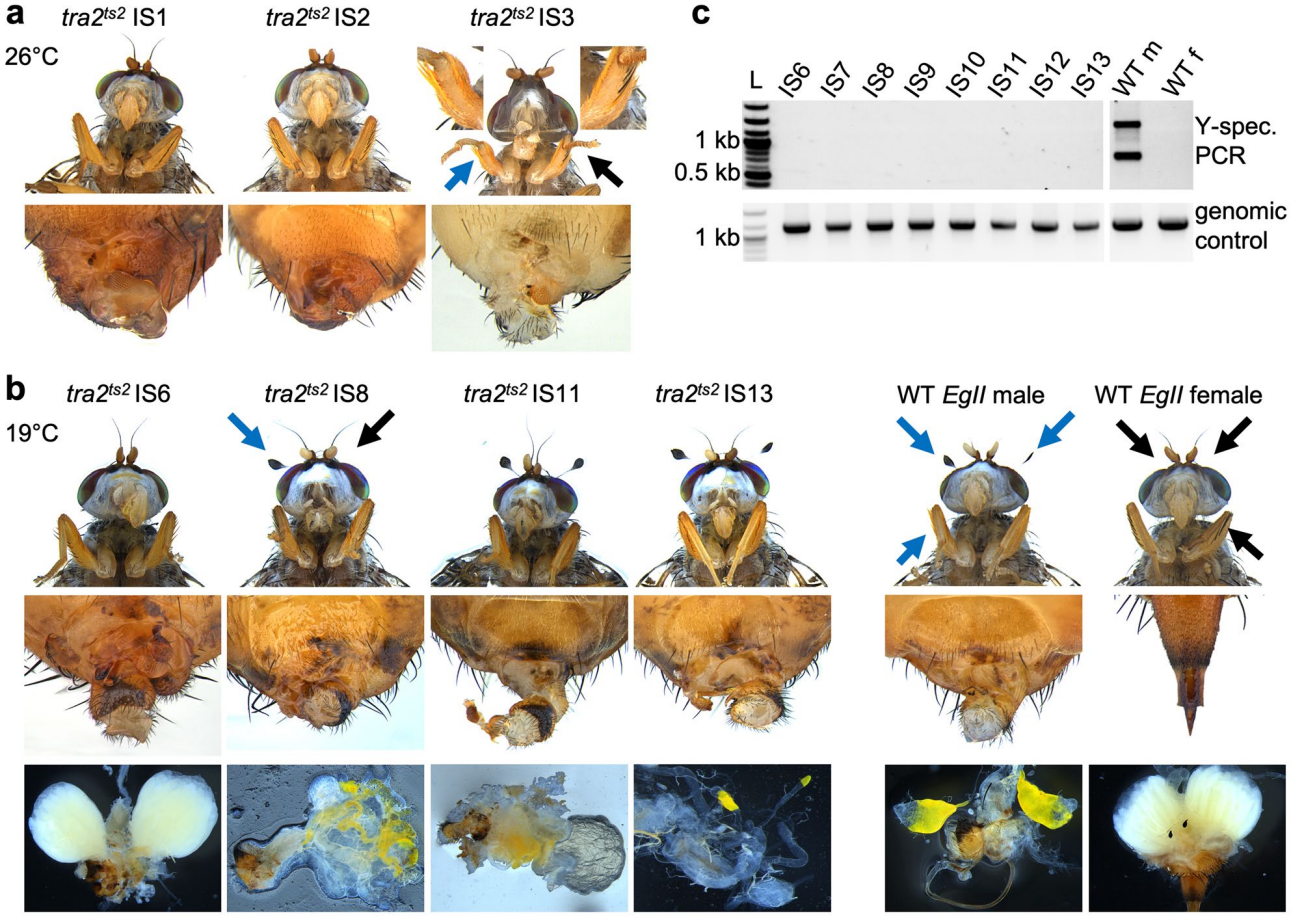
Somatic modification of *tra2*^*ts2*^ causes intersexuality with external and internal phenotypic abnormalities in G_0_. G_0_ survivors of *tra2*^*ts2*^ KI injections reared at 26 °C (**a**) and 19 °C (**b**) show intersex phenotypes with malformed external and internal reproductive organs and mixed male- and female-specific characteristics. Phenotypes included deformed ovipositors (IS1, IS2), a mixture of male- and female-specific bristles on the femur (IS3) or the head (IS8), absent genitalia (IS3), and various degrees of deformed male genitalia, combined with ovaries without spermatheca (IS6), testes-like structures (IS8, IS13) or no identifiable reproductive organs (IS11). For comparison, wild-type males have two spatulated bristles on the head, non-pigmented bristles on the femur, and male genitalia. Wild-type females have no spatulated bristles on the head, long pigmented bristles on the femur, and a prominent ovipositor. Male characteristics are highlighted by blue arrows, female characteristics by black arrows. **(c)** Karyotyping via Y-chromosome-specific PCR of intersex phenotype *tra2*^*ts2*^ KI individuals (19 °C) revealed XX-karyotype for all intersex individuals. Shown is the Y-chromosome-specific PCR (primers P1504/1505) on genomic DNA extracted from a single fly and a genomic positive control on *tra2* with primers P1401/1500 using the same DNA samples. Wild-type male (WT m) and female (WT f) are shown as control. Displayed are cropped parts from different gels. Uncropped versions of the gels are provided in the supplement (Supplementary Fig. S4). L = DNA ladder; kb = kilo base pairs.

The *tra2*^*ts2*^ gRNA knock-out injection yielded six adult males from 367 injected *EgII* embryos (Table 1b), three of them were fertile. Additionally, three G_0_ flies stuck in the puparium did not survive (two males, one intersex IS1-KO). The *tra2*^*ts2*^ knock-in mix was injected into 244 *EgII* embryos (Table 1b). Eight developed to adults (six males, two intersex: IS1, IS2), and four died during eclosing (one male, three intersex: IS3-6). Intersex flies showed varying degrees of phenotypically male and female characteristics (genital terminalia apparatus and bristles) (Fig. 2a), and were sterile. In contrast, all six G_0_ males were fertile. The genotype of six *ts2* G_0_ flies from the knock-out (males M5, M6, and intersex IS1-KO) and knock-in injection (IS1, IS4, IS5) was analysed. All showed NHEJ events ranging from 33 bp deletions to 4 bp insertions (Supplementary Fig. S1b). G_1_ offspring from both injections was not analysed.

These experiments confirmed the editing activity of the *ts1* and *ts2* gRNAs. The lack of fertile G_0_ in the *ts1* injections and the complete lack of females and appearance of intersexes in the *ts2* injections, however, indicated that 26 °C is a restrictive temperature for the *Cctra2*^*ts*^ mutations.

### Evaluation of medfly rearing at low temperatures

To evaluate if *D. melanogaster* and *D. suzukii tra2*^*ts2*^ permissive temperatures, 16 °C or 16 to 20 °C, respectively^30,31,38^, are applicable to medfly, newly eclosed WT *EgII* (60–160 adults per experiment) were transferred from 26 °C to 16, 18, or 19.5 °C and eggs of these crosses were collected for seven days (for temperature profiles and egg collection timepoints see Supplementary Table S1 and Fig. S2a–c). At 19.5 °C, the number of adult offspring was reduced to about 40%, compared to 26 °C, and at 18 °C to about 1%. At 16 °C, no larvae hatched from more than 2,000 collected eggs. Hence, 19 °C was chosen as a possible rearing and potential permissive temperature for the subsequent *Cctra2*^*ts*^ injections.

### CRISPR/Cas9-HDR injections at 19 °C do not produce stable *Cctra2*^*ts1*^ lines

Rearing of *ts1*-injected G_0_ at 19 °C increased the number of adult G_0_ survivors to 19.5% compared to 3% and 2.4% for the *ts1* injections at 26 °C (Table 1a). None of them showed external phenotypic abnormalities. Twelve G_0_ flies were backcrossed individually to *EgII* (M5-M10 and F4-F9), remaining flies were backcrossed in three groups (M-group I, F-group I, II). After allowing sufficient time for mating and egg laying, all individually crossed G_0_ flies were dissected to examine their reproductive organs. Phenotypes included females without ovaries (F4), only one ovary (F7), or normal ovaries (F6, F8, F9). Males showed normal reproductive organs, except for M7, which had no testes (Supplementary Fig. S3a, b). F5 and M10 died and could not be dissected. Overall, 47 G_1_ flies eclosed from eight fertile families (F6, F8, M6, M8, M9, M10, group M_I and F_II). Since no phenotypic marker was inserted to track successful mutagenesis in G_1_, non-lethal genotyping was used to analyse G_1_ offspring reared at 19 °C for the presence of the *tra2*^*ts1*^ mutation. DNA was extracted from a single leg, and the *ts1* target site region was PCR-amplified and sequenced. 38 of 47 G_1_ flies provided sufficient quality sequence information. All showed WT genotype.

### CRISPR/Cas9-HDR successfully creates inheritable *Cctra2*^*ts2*^ mutation at 19 °C

Rearing of *ts2* HDR-injected G_0_ at 19 °C yielded lower survival numbers than the *ts1* HDR injection, but still about twice as high as the experiments at 26 °C (7.1% compared to 2.4% and 4.9%; Table 1b). Injection of 181 *EgII* embryos resulted in five viable males and six intersex. Additionally, two intersex flies (IS9, IS10) died during eclosing. Males and intersex were individually backcrossed to WT virgin females. Eggs were collected every second day for 10 consecutive days (for temperature profile during egg collection see Supplementary Table S1, Fig. S2b). Two of the eleven crosses (M8, M11) produced G_1_ offspring (Supplementary Table S2). After mating, all alive G_0_ were dissected. Males M8, M9, and M11 showed normal testes, while M10 did not have testes (Supplementary Fig. S3c). Flies with intersex phenotype showed apparently normal ovaries but no spermathecae (IS6), hypertrophic testes (IS8), miniaturized testes (IS13), or no identifiable reproductive organs (IS7, IS11, IS12; Fig. 2b). To assess the karyotype of all 13 G_0_ flies, PCR on Y-chromosome-specific repetitive elements was performed, whereby absence of a PCR signal implies a XX-karyotype. None of the intersex phenotype G_0_ flies was positive for the Y-chromosome-specific PCR (Fig. 2c), indicating that all XX (female) karyotype G_0_ embryos were transformed to intersex flies. The absence of phenotypically female G_0_ in all three *tra2*^*ts2*^ injections indicates a high efficiency of gRNA_tra2_ts2 and the importance of the targeted position for proper TRA2 function in female sex development.

The *tra2* genotype of the G_1_ flies was analysed via non-lethal genotyping. For family M8, ten of twelve analysed G_1_ (83%) were heterozygous for the knock-in genotype (*tra2*^*ts2*^), and two (17%) carried NHEJ events. The remaining individuals were not analysed due to low DNA quality. From the G_1_ offspring of family M11, 60 flies were randomly chosen for genotyping. The heterozygous *tra2*^*ts2*^ genotype was found in 45 flies (75%). This percentage was similar in males (26 of 33) and females (19 of 27). Nine flies (15%) were WT and six flies could not be analysed (low DNA quality).

### Inbreeding of the *ts2* mutation at 19 °C does not produce phenotypic females homozygous for *tra2*^*ts2*^

**Figure 3:**
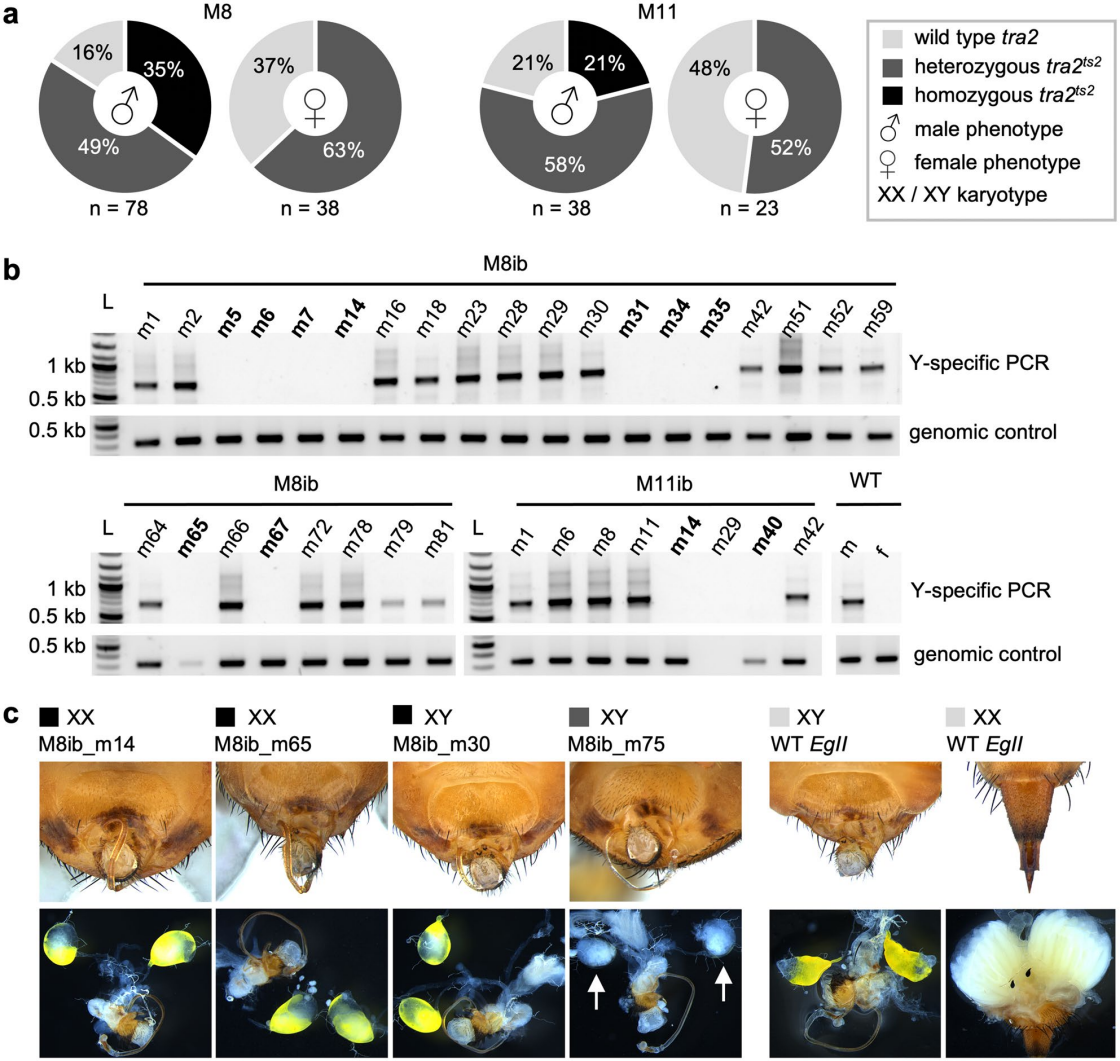
Analysis of *tra2*^*ts2*^ genotypes and phenotypes in G_2_. (**a**) Shown are frequencies of *tra2* genotypes (homozygous for the WT or the *tra2*^*ts2*^ allele, or heterozygous *tra2*^*ts2*^ mutants) within the number of analyzed individuals (n), found in phenotypic male or female G_2_ offspring of family M8 and M11 inbreeding (ib). Both families are lacking homozygous *tra2*^*ts2*^ mutants with a female phenotype. (**b**) Karyotyping of phenotypic G_2_ males via Y-chromosome specific PCR (primers P1504/1505) on genomic DNA extracted from a single leg of family M8 and M11 offspring. A positive control PCR was performed on *tra2* with primers P1532/P1500 using the same DNA samples as in the Y-specific PCR, to exclude lack of PCR product due to DNA quality. Individuals lacking a signal in the Y-chromosome-specific PCRs but not in the genomic control PCR are marked in bold letters to indicate the XX-karyotype. M11ib_m29 was excluded from the analysis, due to low DNA quality. One phenotypic male (WT m) and female (WT f) from family M8 with WT *tra2* genotype are shown as controls. Displayed are cropped parts from different gels. Uncropped versions of the gels are provided in the supplement (Supplementary Fig. S5a and b). L = DNA ladder; kb = kilo base pairs. (**c**) Phenotypic male flies carrying the *tra2*^*ts2*^ mutation were dissected and compared to WT *EgII* flies to assess external and internal sexual organ formation. Shown are representative *tra2*^*ts2*^ homozygous XX (M8ib_m14, M8ib_m65) or XY (M8ib_m30) individuals as well as one XY male heterozygous for *tra2*^*ts2*^ (M8ib_m75). Black, dark and light grey boxes indicate the *tra2* genotype, with colors following the legend in (a). Mutants were not able to coil and store their distiphallus. Testes were normal or decolorized (M8ib_m75).

Heterozygous *tra2*^*ts2*^ mutant G_1_ flies were either inbred or backcrossed to *EgII* to ensure the propagation of the line if inbreeding should turn out to be sterile. Details on crosses, egg collection numbers, and temperature profiles are shown in Supplementary Tables S1, S3, and Fig. S2. Inbreeding of heterozygous M8 offspring produced 121 G_2_ flies with a 1:2 female to male ratio (Supplementary Table S3). 27 of 78 phenotypic G_2_ males were homozygous for the *ts2* mutation (*tra2*^*ts2|ts2*^), 38 were heterozygous (*tra2*^*ts2*|WT^), and 13 were WT (*tra2*^WT|WT^, Fig. 3a). In contrast, none of the 38 phenotypic females were homozygous for *tra2*^*ts2*^, 24 were heterozygous, and 14 had two WT *tra2* alleles (Fig. 3a). *Inter se* crosses of M11 offspring resulted in a similar phenotypic female to male ratio as M8 inbreeding (26 and 42, respectively). Non-lethal genotyping showed that also M11 inbreeding produced phenotypic *tra2*^*ts2*^-homozygous males (21%), but no phenotypic females with two *tra2*^*ts2*^ alleles (Fig. 3a). Backcross of *tra2*^*ts2*^ heterozygous M11 offspring produced a 1:1 phenotypic sex ratio (Supplementary Table S3), which was not further analysed molecularly.

### *tra2*^*ts2*^ homozygous XX embryos are transformed into phenotypic males at 19 °C

The absence of phenotypic females homozygous for the *tra2*^*ts2*^ mutation in G_2_ implied that XX embryos homozygous for *tra2*^*ts2*^ are either not viable or transformed into phenotypic males at 19 °C. Y-specific primers were used to assess the karyotype of 35 G_2_ *tra2*^*ts2*^*-*homozygous and 60 heterozygous male G_2_ flies by PCR. In family M8, nine of 27 phenotypic males homozygous for *tra2*^*ts2*^ showed a signal in the control genomic PCR but not in the Y-chromosome-specific PCR, confirming the transformation of *tra2*^*ts2-*^homozygous XX flies into phenotypic males. This also applied to two out of eight phenotypic males in family M11 (Fig. 3b). For one M11 offspring, M11ib_m29, no statement can be made as the control PCR failed to produce a signal. In contrast, all *tra2*^*ts2-*^heterozygous males were positive for the Y-chromosome-specific PCR (Supplementary Fig. 6), excluding sex conversion as reason for the male-biased sex ratio in the G_2_ heterozygotes.

Dissection of six XX- and four XY-karyotype males homozygous for *tra2*^*ts2*^, and two XY *tra2*^*ts2*^-heterozygous males (all G_2_) showed that all *tra2*^*ts2*^-homozygous males (XX and XY) had apparently normal or slightly decolorized testes. The two *tra2*^*ts2*^-heterozygous males, in contrast, showed severely decolorized testes (Fig. 3c). In addition, across the G_2_ offspring of both families, M8 and M11, 81.8% of the *tra2*^*ts2*^ homozygous XX males, 4.3% of the *tra2*^*ts2*^ homozygous XY males, and 16.6% of the *tra2*^*ts2*^ heterozygous XY males were not capable to coil and store their distiphallus (Fig. 3c). This phenotype was also observed in random samples of WT flies of different ages; while its penetrance in WT is higher at 19 °C (24.8%, n = 161) than at 26 °C (6.9%, n = 174), it is still markedly lower than observed in the *tra2*^*ts2*^ homozygous XX males (81.8%, n = 11) and might, therefore, be also an effect of the *ts2* mutation.

### Rearing at lower temperature leads to low fertility rates

Based on the karyotyping experiments, 19 °C still is a restrictive temperature for the *ts2* mutation in *Cctra2*, contrary to *D. suzukii tra2*^*ts2*^ where 20 °C was permissive^38^. Data from *D. melanogaster* suggests 16 °C as permissive temperature^30,31^. However, medflies do not breed at such low temperatures, as the small-scale fertility tests at 16 °C had shown. To attain a permissive temperature for the medfly *tra2*^*ts2*^ mutation that does not affect breeding, the temperature was lowered to 18.5 °C, the mating threshold temperature^41^, for G_2_ crossing and egg laying (Supplementary Table S1, Fig. S2c). *ts2*-homozygous XX and XY G_2_ males were backcrossed to *EgII* females individually (13 crosses) or in groups (two crosses). *ts2*-heterozygous males and females were inbred (three crosses) or backcrossed (one group). Overall, during 13 days and 81 egg collections, more than 8,000 eggs were collected from these 19 crosses (Supplementary Table S4). A total of five larvae hatched from two egg collections of homozygous *tra2*^*ts2*^ XY male group-backcrosses, and only one survived to adulthood (M11ib_m1-het, Supplementary Table S4). Noteworthy, due to technical restrictions the temperature could not be kept constantly at 18.5 °C during the experiment, and these larvae hatched from a late egg collection (383 h; Supplementary Fig. S2c), prior to which the temperature had been above 18.5 °C for about two days. The male (G_3_) was crossed to 40 *EgII* females but did not reproduce. Therefore, maintaining the *ts2* mutant strain by lowering the temperature to a permissive range was not possible.

## Discussion

CRISPR/Cas9-HDR gene editing was used to create temperature-sensitive mutations in the *C. capitata* sex-determination gene *transformer-2*, equivalent to the two chemically induced point mutations in *D. melanogaster*^30,31^. The *D. melanogaster tra2*^*ts*^ temperature-dependent sex-conversion phenotype promises great advantages for creating male-only populations needed for SIT programs, as it doubles the amount of male offspring per parental egg capacity, and only heat is needed for induction. Some countries do not regulate the use of organisms carrying CRISPR-induced SNPs as they could have also occurred by natural mutagenesis and selective breeding^40,42^. Hence, only the *tra*^*ts*^ SNPs, but no exogenous DNA was inserted, to help facilitate a potential field release of *Cctra2*^*ts*^ strains. This was possible due to the high mutagenesis rate achieved with our previously published CRISPR/Cas9-HDR protocol^39^, which we now successfully applied for the first time without using a visible phenotype.

The injections aiming at creating the *tra2*^*ts1*^ allele did not result in any mutant G_1_ offspring at 19 °C, despite promising prerequisites; *ts1* gRNA and ssODN were functional in the preliminary tests at 26 °C, and the high number of G_0_ adult survivors in the 19 °C injection increased the chance to obtain mutant offspring. Moreover, G_0_ flies showed deformities of internal reproductive organs (Supplementary Fig. 3b). It can’t be excluded, however, that these are the result of physical damage to the embryo caused by the injection. Possible reasons for the poor efficiency of the *ts1* knock-in could be the low on-target activity score of the *ts1* gRNA (0.045), or a stronger phenotypic impact of the *tra2*^*ts1*^ mutation compared to *tra2*^*ts2*^ as observed in *D. melanogaster*^30^, which could reduce the chance to obtain viable *ts1* mutant flies. Testing of other *ts1* gRNAs could shed more light on possible reasons for the failure to create a stable *ts1* line; but considering the decreased viability in *D. melanogaster* and the permissive temperature issues in medfly, these experiments have little prospect for success.

In contrast, the *tra2*^*ts2*^ mutation could be introduced with high efficiency, detectable already from the absence of phenotypic females and the appearance of intersexes in G_0_, in the frequency of HDR-positive fertile G_0_ (100% at 19 °C), as well as in the high penetrance of the mutant genotype within their G_1_ offspring (83% and 75% knock-in for family M8 and M11, respectively). This matches the higher on-target activity score of the *ts2* gRNA (0.140).

The observed overall higher survival rate of injected G_0_ at 19 °C compared to 26 °C might be the result of a lower Cas9 editing activity^43^ and a potentially associated off-target rate, but could also be connected to the reduced speed of embryonic development allowing more time for repair mechanisms to fix injection-induced damage to the embryo^44^, which is unrelated to Cas9 editing. Extensive comparative injections would be needed to answer this question.

The lack of phenotypic females homozygous for *tra2*^*ts2*^ and the conversion of XX embryos into phenotypic males at 19 °C suggests that this is still a restrictive temperature for the *Cctra2*^*ts2*^ mutation, which does not allow correct protein folding, and indicates the importance of this position in the highly conserved TRA2 linker region for correct protein conformation. This observation is in line with the results obtained for the *D. melanogaster tra2*^*ts2*^ mutation, where the temperature had to be lowered to 16 °C to generate fertile males and females, while 18 °C produced sterile males and females, and 29 °C resulted in sterile males and pseudomale-like intersexes^30^. A further reduction of the temperature to an average of 18.3 °C, however, resulted in a loss of the strain due to mainly unviable eggs deposited by the G_2_ generation. This was not unexpected since our small-scale tests with WT at 18 °C and 16 °C produced very little or no viable offspring, respectively. Furthermore, some males were not capable of coiling and storing their distiphallus. While this phenotype was also observed in WT males at low temperatures, it seems to be enhanced by the *tra2* mutant allele. However, the numbers are too small for a robust statement. Fertility and mating behaviour of this phenotype have not been assessed.

While the mean survivorship of medfly egg and larval stages at 15, 20, 25 and 30 °C has been reported to not differ significantly^21^, and the described threshold for ovarian maturation with 8.1 °C to 16.6 °C^21,41,45^ is also below the tested *Cctra2*^*ts2*^ permissive temperature of 18.3 °C, Prokopy and Hendrichs^41^ showed that 18.5 °C is the temperature threshold for mating in medfly. During the cross of *tra2*^*ts2*^ G_2_ flies, temperatures were above the threshold mainly during the first days (1–72 h) and last days (337–517 h) of the crossing (Supplementary Table S1, Fig. S2c). As ovarian maturation takes up to 10 days at this temperature, and crosses have been set up with 3–5 d old flies, no successful mating could have been achieved during the first period above 18.5 °C. During the main egg collection period (72–336 h), temperature was mainly below 18.5 °C (Supplementary Fig. S2c). The successful mating appeared within the second period of exceeded temperature. A possible explanation for the loss of the *tra2*^*ts2*^ strain therefore is that the low temperatures prevented mating and eggs have not been fertilized until temperatures had exceeded 18.5 °C for at least 2 d. On the other hand, control crosses of *EgII* flies managed to produce a small amount of offspring at temperatures mainly lower than or equal to 18.5 °C (2,796 collected eggs, 16 larvae, 8 adults; Supplementary Fig. S2c), showing that low mating activity is taking place at or below the threshold. Therefore, it is possible that the *ts2* mutation, even in the heterozygous state, affects the fertility of the flies at temperatures lower than 18.5 °C. However, as numbers are very small, no robust statement is possible. Overall, using the *EgII* background for the *tra2*^*ts*^ experiments, it could not be determined if the permissive temperature for the medfly *tra2*^*ts2*^ mutation is lower than 18.5 °C or if the *ts2* mutant phenotype in medfly is not temperature-dependent at all.

As strains with different genetic backgrounds can have markedly different sensitivities for elevated or low temperatures due to adaptation mechanisms, using another medfly WT background might allow to investigate lower permissive temperatures for *ts2*. It might also be possible to induce cold acclimation in a WT strain by successively reducing the rearing temperature over several generations before generating the *tra2*^*ts2*^ mutation. This strategy would fail, however, if there is no acclimation with respect to the mating threshold, as shown for *B. tryoni*^46^*.*

Moreover, with regard to the use of the *tra2*^*ts2*^ mutation for medfly sexing in a mass-rearing facility, the presumably low (< 18.5 °C) permissive temperature of the medfly *ts2* mutation would be problematic, as temperature and development time show a linear relation. At 19 °C, for example, the development from egg to adults takes about 32.7 d plus 9 d for ovarian maturation, compared to 17.4 d plus 5.3 d at 26 °C^21^. The even longer development times at < 18 °C would thus be problematic for the production scale and the cost-effectiveness of a mass-rearing program and investigations into lower temperatures would thus certainly not be relevant for insect pest control applications in medfly.

In conclusion, we demonstrated the successful creation of the *D. melanogaster tra2*^*ts2*^ point mutation in *C. capitata* via markerless CRISPR/Cas9-HDR gene editing and the importance of the respective amino acid for the correct function of TRA2 in the female sex-determination. The previously shown high HDR efficiency in medfly using a ssODN repair template to convert the marker gene eGFP (enhanced green fluorescent protein) into BFP (blue), could be confirmed in this study, where we achieved 100% knock-in efficiency (2 out of 2 fertile G_0_) compared to 86% (6 out of 7 fertile G_0_) in the previous study^39^. Also, the high penetrance of mutant offspring within the G_1_ with 75–83% in this study is similar compared to 90% in the previous one. It was not possible, however, to identify a permissive temperature at which the tra2^*ts2*^ mutation does not affect female development, as it would be located below the mating threshold of medfly. Therefore, it could not be determined if we hadn’t reached the permissive temperature yet, or if the *tra2*^*ts2*^ phenotype in medfly, in contrast to *Drosophila,* is not temperature dependent. Based on the data presented here, a medfly sexing strain built solely on *tra2*^*ts2*^ would be unsuitable for an SIT program and mass-rearing, either because the rearing would be too slow to be productive on a large scale, or because the sex conversion could not be switched off for strain maintenance. Other possibilities to create a sex-conversion system in medfly could be to target other sex-determination genes, like *transformer*^27,29,47^, or to force (over)expression of the maleness factor *MoY*, which induces masculinization in XX embryos^48^. However, conditionality would need to be engineered for both options.

## Material and methods

### Rearing conditions

*Ceratitis capitata* wild-type *Egypt-II* (*EgII*) flies were received from the FAO/IAEA Agriculture and Biotechnology Laboratory, Austria, and kept at 26 °C, 48% RH and 14/10 h light/dark cycle. For fertility tests, freshly eclosed *EgII* adult flies were transferred from 26 °C to 19.5 °C, 60% RH, 24 h light or 16 or 18 °C, 46–48% RH, 24 h light, where egg collections and subsequent rearing took place. *tra2*^*ts*^ mutants were kept at 19 °C or 18.5 °C, 60% RH, and 24 h light. Temperature and humidity were measured every five minutes of the experiment using an EL-USB-2 data logger (Lascar electronics, measurement precision for temperature ± 1 °C, for humidity ± 3%). Readout of the data logger showed that during the rearing of the *tra2*^*ts2*^ mutants, short-term variations of the temperature (+ 3 °C/− 1 °C) occurred (see Supplementary Table S1 and Fig. S2). These could not be avoided due to technical restrictions of the experimental setup. Furthermore, the targeted temperature (19 °C) was once exceeded for 3.5 h up to a temperature of 25 °C during an outage of the air conditioning system. This occurred during the late larval or pupal stage of *tra2*^*ts2*^ G_1_. Feeding and screening conditions were as described in Aumann et al.^39^.

### CRISPR/Cas9 gene editing

Design of gRNAs targeting *tra2* (gRNA_tra2_ts1 and gRNA_tra2_ts2) and assessment of potential off-target effects was performed using the *C. capitata* genome version Ccap 2.1 (GCF_000347755.3, NCBI)^49^ and the Software Package Geneious Prime^50^. On-target activity score was 0.045 for gRNA_tra2_ts1, and 0.140 for gRNA_tra2_ts2 (scores are between 0 and 1; 1 = highest expected activity^50^). Both gRNAs showed zero off-targets sites in the medfly genome. gRNA synthesis, in vitro transcription and purification was performed as described before^39^, using primers P_1439 (GAAATTAATACGACTCACTATAGGTGATGATATAGCTGATGCTAGTTTTAGAGCTAGAAATAGC) and P_369 (GCACCGACTCGGTGCCACTTTTTCAAGTTGATAACGGACTAGCCTTATTTTAACTTGCTATTTCTAGCTCTAAAAC) for gRNA_tra2_ts1 and primers P_1440 (GAAATTAATACGACTCACTATAGGCCCATATAAACGCCAGGTGTGTTTTAGAGCTAGAAATAGC) and P_369 for gRNA_tra2_ts2. The sequences of the 140 bp single-stranded HDR templates ‘ssODN_tra2_ts1′ and ‘ssODN_tra2_ts2′ (EXTREMer oligo, Eurofins Genomics) were: ssODN_tra2_ts1 (sense): TGAGTAATCTACGCGTATGCGTCGATCATCGATTTCCATGCCGGAACATGCGTCCTTGGCTGC**T**TTA**A**CATCAGCTATATCATCATAATAGATAAAGCAAAAGCCACGAGATCGGCCAGTCTGAAAAAAGAAAAAAATAG; ssODN_tra2_ts2 (antisense): AAACGATTTAAATCACATGCACATGCGAAGTATACCTTGTGTGTCGTCCCATATAAACGCCAGGTGTGG**A**AGTGTGTGGTCTCTGTGTAGTTGAGTAATCTACGCGTATGCGTCGATCATCGATTTCCATGCCGGAACAT; Base changes introducing the *ts1* or *ts2* mutation are shown in bold. Purified Cas9 protein (PNA Bio Inc.) was reconstituted to 1 µg/µl in 20 mM Hepes, 150 mM KCl, 2% sucrose and 1 mM DTT (pH 7.5).

*Microinjection of embryos:* 10 µl injection mix for knock-out experiments contained 360 ng/µl Cas9 protein and 200 ng/µl gRNA_tra2_ts1 or gRNA_tra2_ts2 in 300 mM KCl^39,51^. For knock-in experiments, 200 ng/µl ssODN_tra2_ts1 or ssODN_tra2_ts2 were added to the mix. The mixes were freshly prepared on ice, incubated at 37 °C for 10 min to allow pre-assembly of gRNA-Cas9 ribonucleoprotein complexes and stored on ice prior to injections. For microinjection of WT *C. capitata* embryos, eggs were collected over a 30–50 min period, prepared for injection and handled afterwards as previously described^39^. Injections were performed using siliconized quartz glass needles (Q100-70–7.5; LOT171381; Science Products, Hofheim, Germany), drawn out on a Sutter P-2000 laser-based micropipette puller. Injection equipment consisted of a manual micromanipulator (MN-151, Narishige), an Eppendorf FemtoJet 4i microinjector, and an Olympus SZX12-TTR microscope (SDF PLAPO 1xPF objective). Injection survivors were numbered successively across ts1 injections and ts2 injections, respectively.

### Crossing strategies and dissection of internal reproductive organs

*Crossing of G*_*0*_*:* Each G_0_ adult injection survivor was individually crossed to three *EgII* WT males or virgin females, except for the 19 °C *ts1* knock-in injection. Here, six males and six females were individually backcrossed, the remaining flies were group-backcrossed (five G_0_ males to 15 females, ten G_0_ females to ten WT males, and six G_0_ females to nine WT males). Eggs were collected three to five times, with an interval of one to two days. For the 19 °C knock-in experiments, G_1_ and G_2_ flies (if applicable) were kept individually until their genotype was assessed via non-lethal genotyping.

*Crossing of tra2*^*ts2*^* G*_*1*_: males and females heterozygous for *tra2*^*ts2*^ were inbred. Additionally, heterozygous males were backcrossed (Supplementary Table S3). Eggs were collected six times, with an interval of one to two days.

*Crossing of tra2*^*ts2*^* G*_*2*_*:* phenotypic males and females heterozygous for *tra2*^*ts2*^ were inbred (Supplementary Table S4). Additionally, four *tra2*^*ts2*^ heterozygous XY males, not capable of coiling and storing their distiphallus, were group backcrossed. *tra2*^*ts2*^ homozygous XY males were either backcrossed or crossed with heterozygous *tra2*^*ts2*^ females (Supplementary Table S4). Nine males homozygous for *tra2*^*ts2*^ with XX-karyotype, all not able to coil and store their distiphallus, were individually backcrossed to four females each. Eggs of the G_2_ crosses were collected four to seven times over seven to 13 days (Supplementary Table S4).

*Dissections*: G_0_ flies and single crossed G_2_ flies were allowed to mate for 5–10 days (G_0_) or 7–13 days (G_2_) days. If still alive, they were then dissected to examine their internal reproductive organs.

### Molecular analyses of G_0_ mosaics

To analyse the mosaic genotype of G_0_ flies, DNA was extracted from single flies according to a standard protocol. The target region encompassing the *ts1* and *ts2* mutant sites (1213 bp) was amplified using the *tra2*-specific primers P1401 (TGCTTGGTGGTCCGCAAATA) and P1500 (TGTGCATATACTAAAGGCTCTCCC), 50–100 ng DNA, and the Q5 High-fidelity DNA polymerase (New England Biolabs) according to the manufacturer’s protocol in a Bio-Rad C1000 Touch thermal cycler [98 °C, 1 min; 35 cycles of (98 °C, 15 s; 56 °C, 30 s; 72 °C, 45 s); 72 °C, 2 min]. PCR fragments were purified using the Zymo Research DNA Clean & Concentrator^-5^ kit and subcloned into the pCR4-blunt TOPO vector (Invitrogen) for sequencing. Three to five clones were sequenced using primer mfs13 (TGTAAAACGACGGCCAGT) (Macrogen Europe, Amsterdam) for each analysed fly. Verification of CRISPR-induced mutations from the sequencing results was performed using the Software Package Geneious Prime^50^ by mapping the sequencing results to the *tra2* reference sequence (Gene ID: 101,452,698).

### Non-lethal genotyping of G_1_ and G_2_ flies

To identify the *tra2* genotype of G_1_ and G_2_ flies, non-lethal genotyping was performed using an adapted version of the protocol established by Carvalho et al.^52^. A single leg of an anesthetized fly was cut at the proximal femur using scissors and homogenized in 50 µl buffer (10 mM Tris–Cl pH 8.2, 1 mM EDTA, 25 mM NaCl) for 15 s (6 m/s) using ceramic beads and a FastPrep-24 5G homogenizer (M.P. Biomedicals). 28.3 µl buffer mixed with 1.7 µl proteinase-K (2.5 U/mg) were added and incubated for 1 h at 37 °C. The reaction was stopped 4 min at 98 °C. The solution was cooled down on ice and directly used as PCR template to amplify the region surrounding the *tra2* target site. A 25 µl PCR reaction contained Dream*Taq* polymerase and buffer (Life Technologies), dNTPs and the *tra2*-specific primers P1401 and P1500 according to the manufacturer’s instructions, and 3.75 µl template solution in a Bio-Rad C1000 Touch thermal cycler [95 °C, 3 min; 35 cycles of (95 °C, 30 s; 56 °C, 30 s; 72 °C, 1 min); 72 °C, 5 min]. The size of the PCR product (1,213 bp) was verified on an agarose gel. PCR products were purified using the Zymo Research DNA Clean & Concentrator^-5^ kit, sequenced using primer P1500, and subsequently analysed using Software Package Geneious Prime^50^.

### Molecular karyotyping-Y chromosome specific PCR

Y-specific repetitive elements were amplified from genomic DNA extracted either from a single fly (G_0_) or a single leg (G_2_) using the published Y-specific oligonucleotides P1504_Y-spec1 (TACGCTACGAATAACGAATTGG) and P1505_Y-spec2 (GCGTTTAAATATACAAATGTGTG)^53^. 10 µl PCR reactions contained either 50 ng DNA (single fly) or 3.75 µl single-leg DNA template solution, and the Y-specific primers and Dream*Taq* PCR components as described above. PCR cycling conditions (Bio-Rad C1000 Touch) were [95 °C, 3 min; 35 cycles of (95 °C, 30 s; 58 °C, 30 s; 72 °C, 1 min); 72 °C, 5 min]. Absence of a PCR product was interpreted as absence of the Y chromosome (XX-karyotype). The same PCR conditions with primers P1532 (AGTGAAAACGATTTAAATCACATGCAC) and P1500 for genomic DNA extracted from a single-leg, or P1401 and P1500 for DNA extracted from a single fly were used to amplify 328 bp or 1,213 bp fragments, respectively of *tra2* as a positive control PCR to confirm sufficient quality of extracted genomic DNA.

### Equipment and settings for image acquisition

For bright field image acquisition of flies (either dead or anesthetized with CO_2_ and placed on a 4 °C cooler) was carried out using a fully automated Leica M205FC stereo microscope with a PLANAPO 1.0 × objective, a Leica DFC7000 T camera and the Leica LAS X 3.4.2.18368 software. To enhance screen and print display of the pictures the image processing software Fiji ImageJ Version 2.0.0^54^ was used to apply moderate changes to image brightness and contrast. Changes were applied equally throughout the entire image and across all images.

## Supplementary information

Supplementary Information.

## Data Availability

All data generated or analysed is included in this article or the supplement.
