# Supplementary material for: Female-to-male sex conversion in Ceratitis capitata by CRISPR/Cas9 HDR-induced point mutations in the sex determination gene transformer-2
Source: Sci Rep. 2020 Oct 29;10:18611. doi: 10.1038/s41598-020-75572-x (PMC7596080; doi:10.1038/s41598-020-75572-x)
Supplement: Supplementary file 1 — Supplementary Information. [file 41598_2020_75572_MOESM1_ESM.pdf]

Supplementary Information for

Female-to-male sex conversion in *Ceratitis capitata* by CRISPR/Cas9 HDR-induced point mutations in the sex determination gene *transformer-2*

Roswitha A. Aumann, Irina Häcker, Marc F. Schetelig*

Justus-Liebig-University Gießen, Institute for Insect Biotechnology, Department of Insect Biotechnology in Plant Protection, Winchesterstr. 2, 35394 Gießen, Germany

*Corresponding author: marc.schetelig@agrar.uni-giessen.de

This PDF file includes:

- Supplementary Methods
- Supplementary Figures S1-S5
- Supplementary Tables S1-S4
- Supplementary References

Supplementary Methods

PCR reactions shown in Supplementary Figure S5 were done in a 10 µl reaction volume containing 3.75 µl single-leg DNA template solution, the *tra2*- or Y-chromosome-specific primers, and Dream*Taq* PCR components according to the manufacturer's protocol. PCR cycling conditions (Bio-Rad C1000 Touch) were [95°C, 3 min; 35 cycles of (95°C, 30 s; 58°C, 30 s; 72°C, 1 min); 72°C, 5 min]. Gel pictures were taken with a VersaDoc MP Molecular Imager (BioRad).

PCR reactions shown in Supplementary Figure S6 were done in a 10 µl reaction volume containing 2.5 µl single-leg DNA template solution, the Y-specific primers and Dream*Taq* PCR components according to the manufacturer's protocol. PCR cycling conditions (Bio-Rad C1000 Touch) were [95°C, 3 min; 40 cycles of (95°C, 30 s; 58°C, 30 s; 72°C, 1 min); 72°C, 5 min]. Gel pictures were taken with a Gel iX imager (Intas, Göttingen).

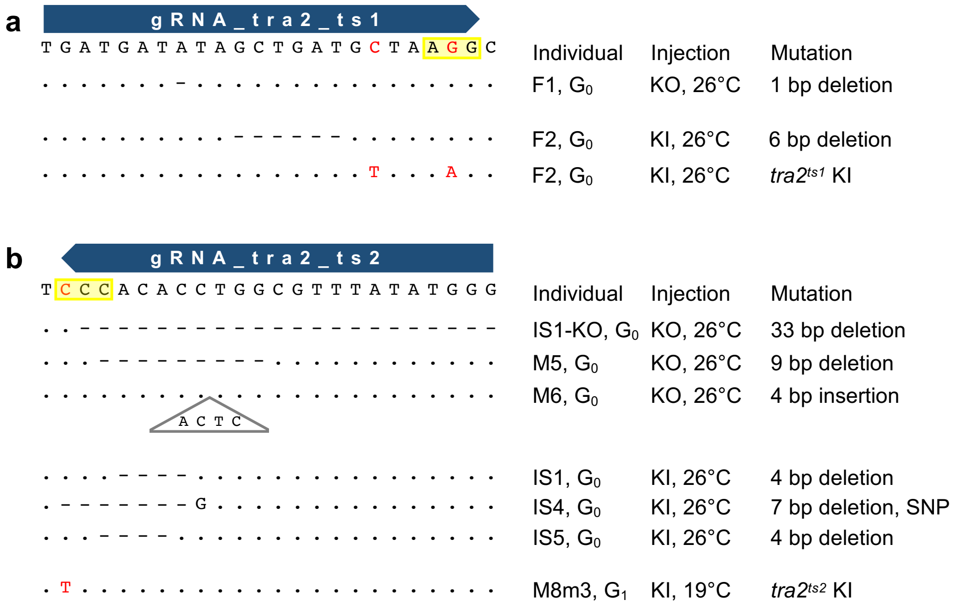

Fig. S1. HDR and NHEJ events confirm ts1 and ts2 gRNA functionality. Sequences of mutant *tra2*^{ts1} **(a)** and *tra2*^{ts2} **(b)** alleles identified in G_0 or G_1 individuals compared to the *tra2* reference sequence. The consensus is shown as dots, knock-in (KI) mutant sites in red uppercase letters, NHEJ induced SNP as uppercase letters, deletions as dashes, insertions as a triangle. The identity of the analysed fly (F = female, M = male, IS = intersex), the injection type (knock-in = KI, knock-out = KO) and rearing temperature, as well as the mutation event are indicated on the right.

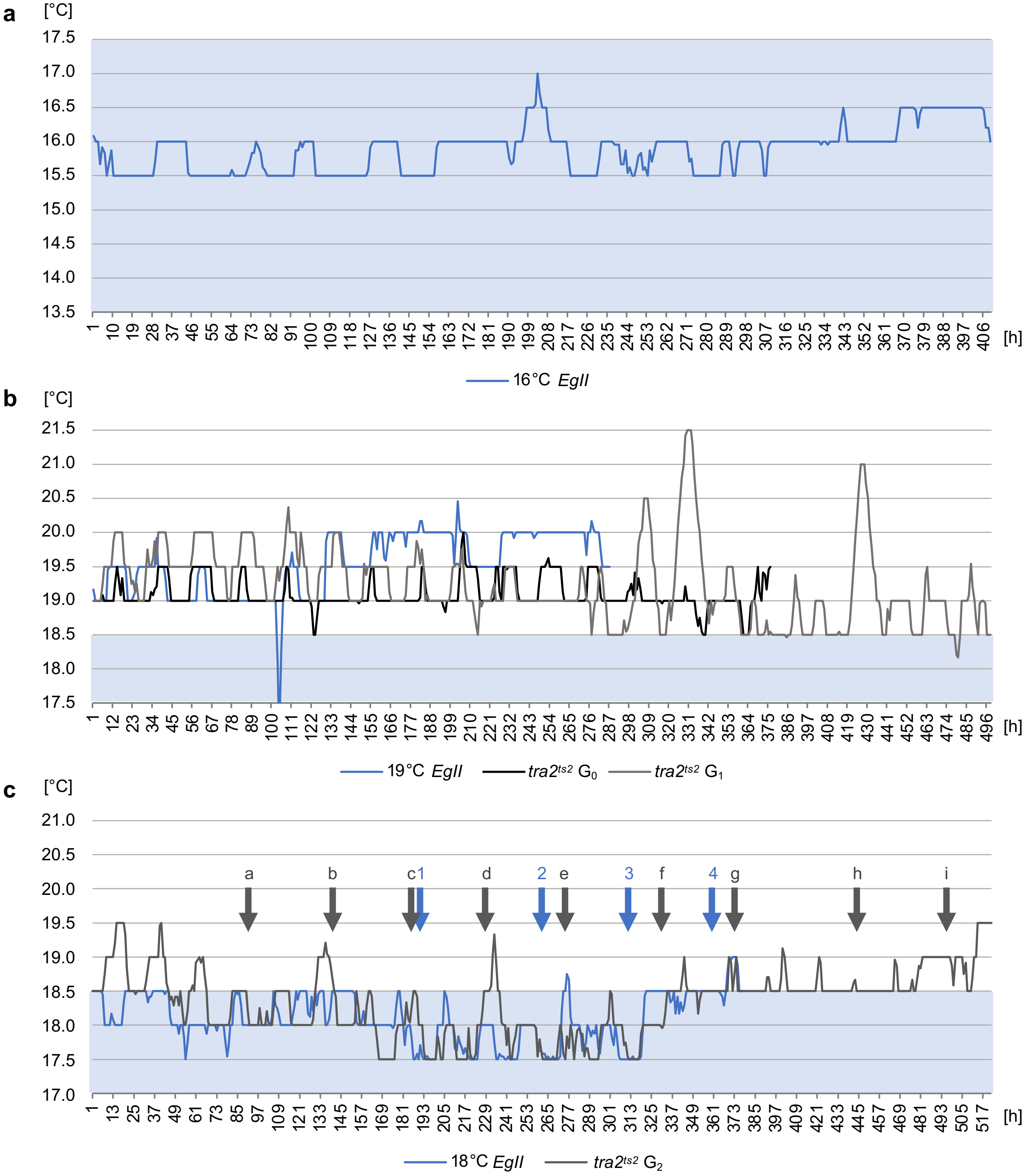

Fig. S2. Temperature profiles during *EgII* control and *tra2*^{ts2} crosses. Shown are the temperature profiles [°C] for the duration of the cross (from set up until the day of the last egg collection, in hours = h), for the wild type control cross at 16°C **(a)**, the cross of *tra2*^{ts2} G_0 and G_1 flies as well as a *EgII* control at 19°C **(b)** and the cross of *tra2*^{ts2} G_2 flies and a control at 18°C **(c)**. Crosses have been set up either with newly eclosed flies (all controls, G_0 cross), or with 3-5 d old flies, if flies needed to be genotyped first (G_1 and G_2 crosses). Temperature was recorded every 5 min. For a clearer visualization of the recordings, the average temperature per hour (average of 12 recording timepoints) is displayed here. Egg collection timepoints of *tra2*^{ts2} G_2 and control crosses are indicated by dark grey (a-i) or blue (1-4) arrows, respectively. For *tra2*^{ts2} G_2, larvae hatched only once, on egg collection timepoint 'g', from two crosses, the group crosses No. 17 and 18 (see Supplementary table S4). For the control, 16 larvae hatched from egg collection timepoints 2, 3 and 4 from overall 2,796 collected eggs. Temperatures below the published medfly mating-threshold (18.5°C; Prokopy and Hendrichs, 1979) are shaded in blue.

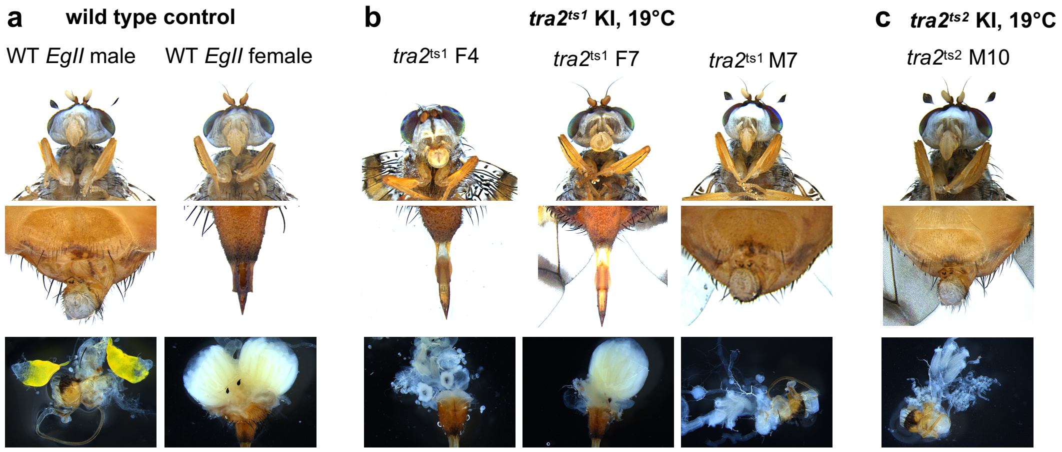

Fig. S3. CRISPR/Cas9 HDR injections at 19°C targeting *tra2^{ts1}* or *tra2^{ts2}* create G_0 flies with abnormal internal sexual organs. Shown are the head, with or without male-specific bristles (top row), the abdomen (middle row), and the internal reproductive organs (bottom row). **(a)** wild type control male and female fly. **(b)** *tra2^{ts1}* KI injection survivors did not show any external abnormalities, however, internal reproductive organs of three out of twelve dissected individuals were distorted; for example, F4 showed no ovaries but spermatheca, F7 had one developed ovary, M7 had no testes (F = female, M = male). **(c)** One out of four dissected male survivors of the *tra2^{ts2}* KI injection (19°C) showed internal abnormalities of sexual organs (no testes).

a Y-chromosome specific PCRs

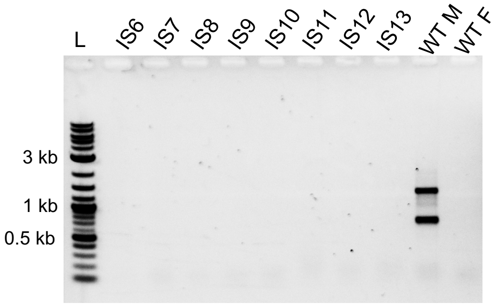

b genomic control PCRs

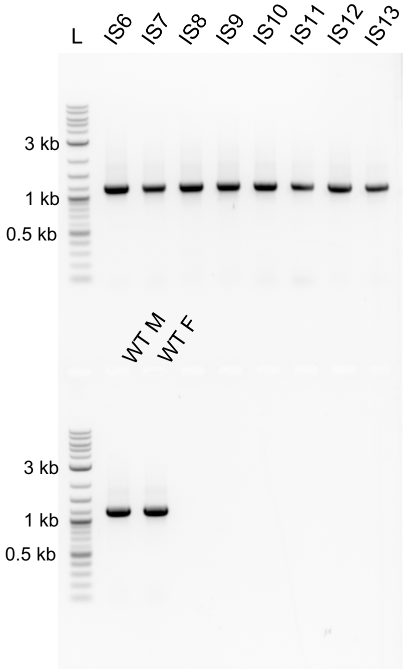

Fig. S4. Karyotyping of *tra2^{ts2}* injected G_0 intersex flies reveals partial masculinization of XX embryos. Uncropped version of the gels shown in Fig. 2c. **(a)** Y-chromosome specific PCR (primers P1504/1505) on genomic DNA extracted from single flies with intersex (IS) phenotype. A wild type *EgII* male (WT M) and female (WT F) served as positive and negative controls, respectively. **(b)** Positive control PCR on the same genomic DNA samples as in a) using primers P1401/P1500 to amplify a 1,213 bp fragment of *tra2.* The DNA ladder (L) used for agarose gels is the NEB 2-log DNA-ladder; kb = kilo basepairs.

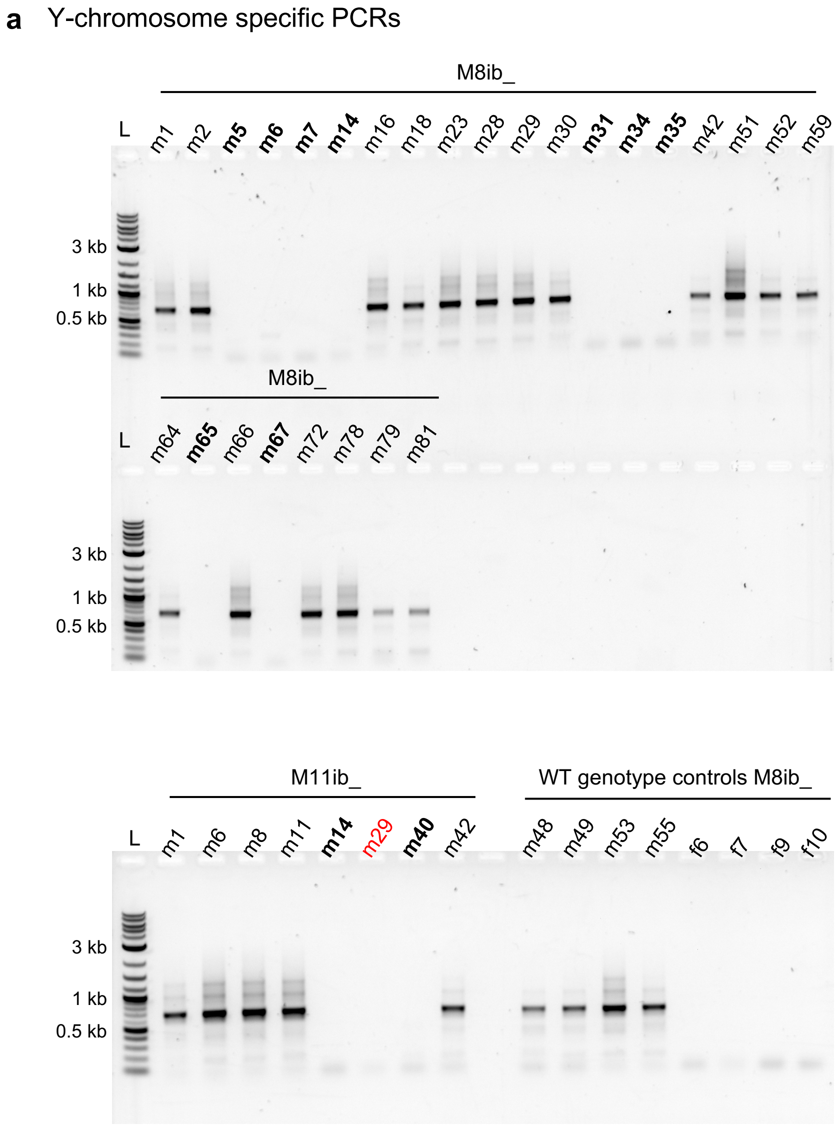

Fig. S5a. Karyotyping of phenotypic G_2 males homozygous for the *tra2*^{ts2} mutation reveals sex transformation of XX embryos. Uncropped version of the gels shown in Fig. 3b. Y-chromosome specific PCR (primers P1504/1505) on genomic DNA extracted from a single leg of family M8 and M11 offspring. Out of 35 analysed *tra2*^{ts2} males, eleven did not show a signal in the Y-chromosome specific PCRs and are marked in bold letters. PCRs were done at least twice to verify the results. M11ib_m29 was excluded from the analysis due to low DNA quality (see Fig. S5 b). Four phenotypic males and females from family M8 with WT *tra2* genotype are shown as controls. The DNA ladder (L) used for agarose gels is the NEB 2-log DNA-ladder; kb = kilo basepairs. A positive control PCR was performed and is shown in Fig. S5b.

b genomic control PCRs

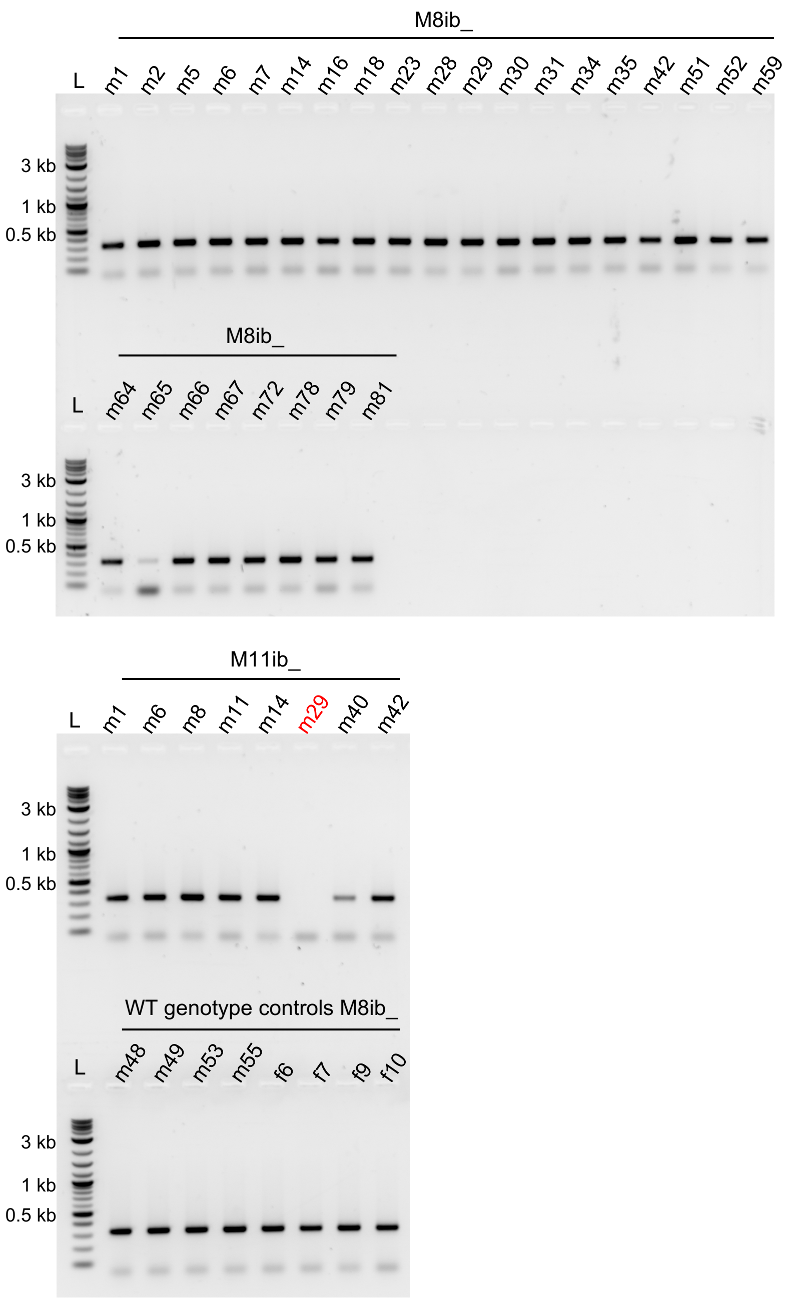

Fig. S5b. Karyotyping of phenotypic G_2 males homozygous for the *tra2*^{ts2} mutation reveals sex transformation of XX embryos. Uncropped version of the gels shown in Fig. 3b. A positive control PCR was performed on *tra2* with primers P1532/P1500 using the same DNA samples as in the Y-specific PCR, to exclude lack of PCR product due to DNA quality. Individuals lacking a signal in the Y-chromosome-specific PCRs but not in the genomic control PCR are marked in bold letters to indicate the XX karyotype. M11ib_m29 was excluded from the analysis due to low DNA quality. Four phenotypic males and females from family M8 with WT *tra2* genotype are shown as controls. The DNA ladder (L) used for agarose gels is the NEB 2-log DNA-ladder; kb = kilo basepairs.

Y-chromosome specific PCRs

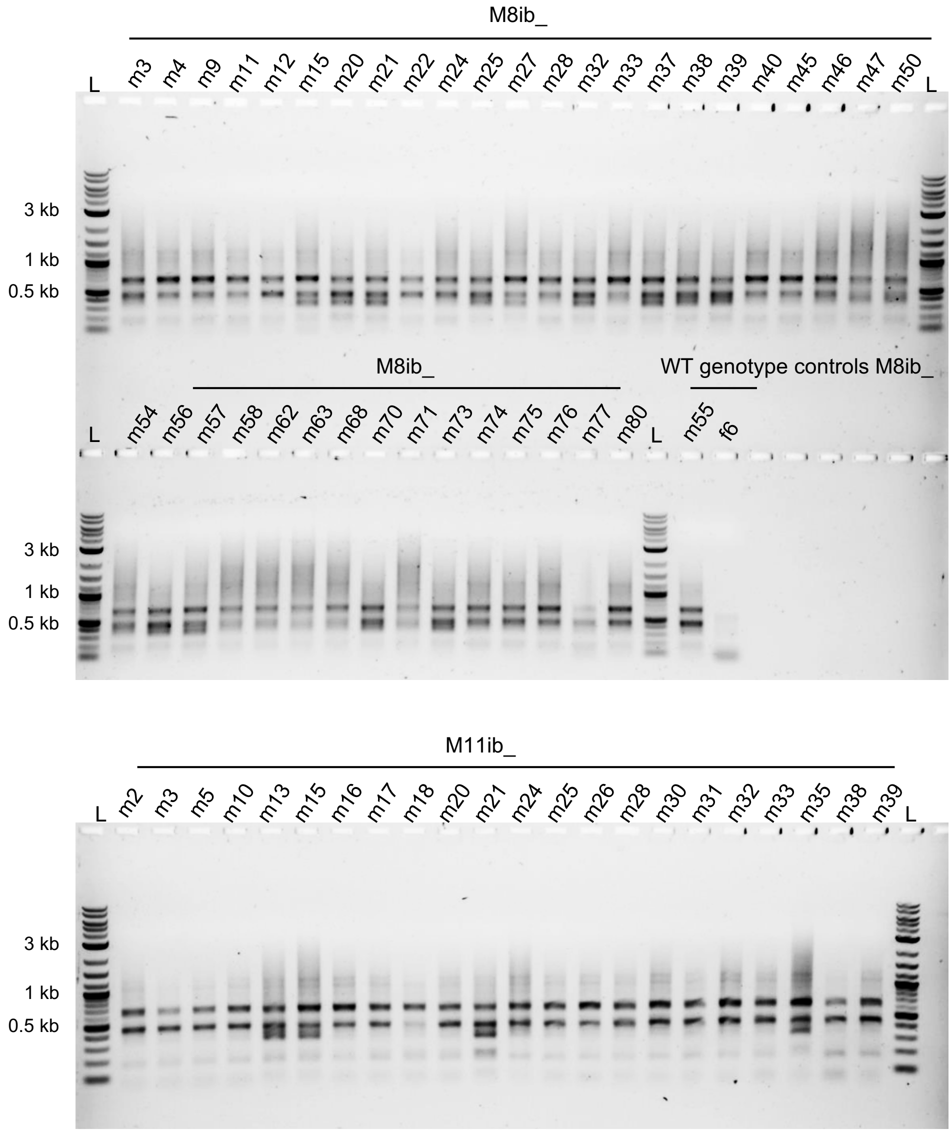

Fig. S6. Karyotyping of phenotypic G_3 males heterozygous for the *tra2*^{ts2} mutation verifies Y-chromosome presence in all individuals. Y-chromosome-specific PCR (primers P1504/1505) on genomic DNA extracted from single legs of *tra2*^{ts2}-heterozygous males, originating from family M8 and M11 inbreeding. One phenotypic male and female from family M8 with WT *tra2* genotype are shown as controls. The DNA ladder (L) used for agarose gels is the NEB 2-log DNA-ladder; kb = kilo basepairs.

Table S1. Temperature profiles during *EgII* control and *tra2^ts2^* crosses. Shown is the cross, the duration of the cross (from setup until last egg collection, in hours = h), the average temperature during this timeframe [°C], the lowest and highest temperature during this timeframe and their durations, and how long the temperature was above the mating-threshold (18.5°C), absolute [h], and in relation to the overall duration [%]. Durations of temperatures are based on the whole timeframe, and their continuity is shown in Supplementary Fig. S2.

**cross**	**duration**	**average temp.**	**min. temp. (duration)**	**max. temp. (duration)**	**temp. ≥ 18.5°C**
16°C *EgII* control	410 h	15.88°C	15.5°C (154.92 h)	17°C (1.75 h)	0 h (0%)
19.5°C *EgII* control	288 h	19.50°C	16.5°C (0.66 h)	20.5°C (2.17 h)	286 h (99.3%)
G_0 cross *tra2*^{ts2}	377 h	19.10°C	18.5°C (12.67 h)	20°C (2.17 h)	376.67 h (99.9%)
G_1 cross *tra2*^{ts2}	498 h	19.20°C	18.0°C (1.33 h)	21.5°C (4.42 h)	496.75 h (99.7%)
18°C *EgII* control	378 h	18.09°C	17.5°C (76.83 h)	19.5°C (0.08 h)	146.67 h (38.8%)
G_2 cross *tra2*^{ts2}	522 h	18.30°C	17.5°C (71.42 h)	19.5°C (17.92 h)	305.83 h (58.6%)

Table S2. Fertile crosses of the *tra2*^{ts2} CRISPR-HDR injection reared at 19°C. Shown are the two fertile G_0 male flies, the number and genotype of females used for backcrossing, the number of G_1 pupae and adult male and female offspring.

**fertile G_0 survivor**	**females: number, genotype**	**G_1 pupae**	**G_1 adults phenotype**
M8	3x WT *EgII*	22	5 females<br>13 males
M11	3x WT *EgII*	96	37 females<br>45 males

Table S3. Crosses of the heterozygous *tra2*^{ts2} G_1 mutants of families M8 and M11 at 19°C. Shown is the number, family, and *tra2* genotype of males and females used to set up the inbreeding and backcrossing cages, the number of eggs collected per cage over all egg collection time points, and the number of G_2 pupae and male and female offspring. Inbreeding crosses are marked in bold.

males: number, family, *tra2* genotype	females: number, family, *tra2* genotype	eggs collected	G_2 pupae	G_2 adults phenotype
**16, M11m, *tra2*$^{ts2/WT}$**	**17, M11f, *tra2*$^{ts2/WT}$**	~ 280	71	26 females<br>42 males
10, M11m, *tra2*$^{ts2/WT}$	22, WT	~ 1.180	53	26 females<br>27 males
**4, M8m, *tra2*$^{ts2/WT}$**	**2, M8f, *tra2*$^{ts2/WT}$**	~ 300	126	38 females<br>82 males

Table S4. Crossing of G_2 *tra2*^{ts2} mutants at 18.5°C. Shown is: in column 1 the numbering of cages (No.), in column 2 the family identifier (M8ib/M11ib), specific name (single cross) or number of flies per cage (group cross), *tra2* genotype, karyotype and ability to coil the phallus of males, in column 3 the number and *tra2* genotype of females, in column 4 the number of eggs collected over all egg collection time points (7-13 days), in column 5 the number of hatched larvae and in column 6 the number of eclosed adults. Inbreeding crosses are marked in bold.

	No.	males: name (single cross) or number and family (group cross), *tra2* genotype, karyotype (XX or XY), unable to coil phallus (P)	females: number, *tra2* genotype	eggs collected	G_3 larvae	G_3 adults
Single cross (backcross)	1	M8ib_m5, *tra2*$^{ts2/ts2}$, XX, P	4, WT	222	0	0
	2	M8ib_m6, *tra2*$^{ts2/ts2}$, XX, P	4, WT	184	0	0
	3	M8ib_m7, *tra2*$^{ts2/ts2}$, XX, P	4, WT	111	0	0
	4	M8ib_m14, *tra2*$^{ts2/ts2}$, XX, P	4, WT	373	0	0
	5	M8ib_m30, *tra2*$^{ts2/ts2}$, XY, P	4, WT	308	0	0
	6	M8ib_m31 *tra2*$^{ts2/ts2}$, XX, P	4, WT	265	0	0
	7	M8ib_m34, *tra2*$^{ts2/ts2}$, XX, P	4, WT	319	0	0
	8	M8ib_m35, *tra2*$^{ts2/ts2}$, XX, P	4, WT	406	0	0
	9	M8ib_m42, *tra2*$^{ts2/ts2}$, XY	4, WT	359	0	0
	10	M8ib_m64, *tra2*$^{ts2/ts2}$, XY	4, WT	343	0	0
	11	M8ib_m65 *tra2*$^{ts2/ts2}$, XX, P	4, WT	478	0	0
	12	M8ib_m67 *tra2*$^{ts2/ts2}$, XX, P	4, WT	399	0	0
	13	M8ib_m81, *tra2*$^{ts2/ts2}$, XY	4, WT	303	0	0
Group cross (backcross or **inbreeding**)	14	4, M8ib_m, *tra2*$^{ts2/WT}$, XY, P	8, WT	500	0	0
	15	**29, M8ib_m, *tra2*$^{ts2/WT}$**	**22, M8ibf, *tra2*$^{ts2/WT}$**	1.743	0	0
	16	**13, M11ib_m, *tra2*$^{ts2/WT}$**	**10, M11ibf, *tra2*$^{ts2/WT}$**	511	0	0
	17	6, M8ib_m, *tra2*$^{ts2/ts2}$, XY	12, WT	751	1	0
	18	4, M11ib_m, *tra2*$^{ts2/ts2}$, XY	8, WT	535	4	1
	19	**2, M8ib_m, *tra2*$^{ts2/ts2}$, XY**	**2, M8ibf, *tra2*$^{ts2/WT}$**	216	0	0

Supplementary references

Prokopy, R. J. & Hendrichs, J. Mating behavior of *Ceratitis capitata* on a field-caged host tree. *Ann. Entomol. Soc. Am.* **72,** 642-648 (1979).
